# A Systematic Review of MRI Neuroimaging for Education Research

**DOI:** 10.3389/fpsyg.2021.617599

**Published:** 2021-05-20

**Authors:** Ching-Lin Wu, Tzung-Jin Lin, Guo-Li Chiou, Chia-Ying Lee, Hui Luan, Meng-Jung Tsai, Patrice Potvin, Chin-Chung Tsai

**Affiliations:** ^1^Program of Learning Sciences, School of Learning Informatics, National Taiwan Normal University, Taipei, Taiwan; ^2^Institute for Research Excellence in Learning Sciences, National Taiwan Normal University, Taipei, Taiwan; ^3^Institute of Neuroscience, National Yang-Ming University, Taipei, Taiwan; ^4^Institute of Linguistics, Academia Sinica, Taipei, Taiwan; ^5^Institute of Cognitive Neuroscience, National Central University, Taoyuan, Taiwan; ^6^Research Center for Mind, Brain, and Learning, National Chengchi University, Taipei, Taiwan; ^7^Département de Didactique, Université du Québec à Montréal, Montréal, QC, Canada

**Keywords:** neuroimaging, MRI, DTI, education research, learning

## Abstract

This study aims to disclose how the magnetic resonance imaging (MRI) neuroimaging approach has been applied in education studies, and what kind of learning themes has been investigated in the reviewed MRI neuroimaging research. Based on the keywords “brain or neuroimaging or neuroscience” and “MRI or diffusion tensor imaging (DTI) or white matter or gray matter or resting-state,” a total of 25 papers were selected from the subject areas “Educational Psychology” and “Education and Educational Research” from the Web of Science and Scopus from 2000 to 2019. Content analysis showed that MRI neuroimaging and learning were studied under the following three major topics and nine subtopics: cognitive function (language, creativity, music, physical activity), science education (mathematical learning, biology learning, physics learning), and brain development (parenting, personality development). As for the type of MRI neuroimaging research, the most frequently used approaches were functional MRI, followed by structural MRI and DTI, although the choice of approach was often motivated by the specific research question. Research development trends show that the neural plasticity theme has become more prominent recently. This study concludes that in educational research, the MRI neuroimaging approach provides objective and empirical evidence to connect learning processes, outcomes, and brain mechanisms.

## Introduction

Educational neuroscience is an emerging field that combines physiology, psychology, cognitive science, developmental science, and education to explore the brain-based foundations of teaching effectiveness and learning mechanisms (Fischer et al., 2010). It has been made clear that mental activity relies on brain functioning (Lalancette and Campbell, 2012). Brain information can provide more direct empirical evidence than information from previous studies, which could only infer the learning process and performance from reaction times, accuracy, and self-reported performance (Ardena et al., 2010). Moreover, educational neuroscience provides an insight to educational thinking through the integration of neural and behavioral data (Howard-Jones et al., 2016). For example, it has been argued that an activation of the function of inhibition can sometimes be recorded when experts produce accurate answers to incongruent problems that contain conceptual traps. However, these experts remain completely incapable of signaling or discussing the presence of interference within their unconscious cognitive deliberation. Thus, using questionnaires or interviews is not useful in the study of such interference. On the other hand, it has been argued that white matter network efficiency can discriminate different types of creative problem solving (e.g., remote association, close association), thus explaining why some people produce original ideas more easily (Wu et al., 2016a). Besides, the brain structure information can also be used as a valid indicator for identifying mathematically gifted and typical development individuals, so that children's learning potential can be discovered at an earlier age (Kuo et al., 2019). Hence, through the understanding of the brain mechanism, the integration of the neuroscience research, learning, and teaching can possibly optimize learning outcomes.

Magnetic resonance imaging (MRI) in neuroimaging research is a part of the general educational neuroscience approach, and includes functional MRI (fMRI), structural MRI (sMRI), and diffusion tensor imaging (DTI) (Shenton et al., 2012). MRI provides relatively high spatial resolution and non-invasive observation of neural activity, including changes in brain oxygen levels (Wu et al., 2019), brain volume (Steen et al., 2006), brain connectivity, and cortical thickness (Pyatigorskaya et al., 2014). fMRI was used to examine the blood-oxygenation level-dependent response, while sMRI was used to analyze the structural properties of the brain (Tan et al., 2013). For example, fMRI was conducted to explore the core brain region involved with representing changes during insight problem solving (i.e., the superior temporal gyrus; Wu et al., 2019); sMRI analyzed the differences in brain structure (i.e., cortical thickness or volume) in different groups (Wu and Kuo, 2020). Furthermore, DTI can examine the diffusion of water molecules inside brain tissue, thereby providing a structural image of brain tissue with nerve fiber orientation (Sporns et al., 2004; Achard et al., 2006; Hagmann et al., 2008; van den Heuvel et al., 2008; Gong et al., 2009). For instance, creative problem solving (i.e., remote association) and humor related traits (e.g., humor preference, fear of being laughed at) were all found to be significantly associated with the integrated connectivity of the brain network via the DTI approach (Wu et al., 2016a,b; Wu et al., 2018). These different neuroimaging observations help us understand how the operation of specific regions in the brain connects to certain cognitive functions, and the correlation between anatomical properties and behavior.

As mentioned previously, the use of MRI neuroimaging approaches in educational research has been growing in recent years. However, most studies have been conducted in medicine (Mouchlianitis et al., 2016; Andryszak et al., 2017). Accordingly, what educational issues have been explored via MRI neuroimaging approaches and how different brain image observations can inform us about cognitive activities during learning are still unclear. Therefore, this study aims to review the applications of MRI for educational research to understand the research topic, research method, and trends, as well as the age level of participants within these MRI studies. We reviewed the literature published in the last 20 years (since 2000); we systematically reviewed literature on sMRI, fMRI, and DTI to explore the different educational topics they dealt with, the developmental stage of the participants, and the brain activation areas involved in various mental functions. The analysis and integration of these data help to construct a framework of how the MRI neuroimaging methods can inform us about the brain operations in relation to educational problems and challenges, thus providing a reference for further educational research and practice.

## Method

### Paper Selection

We conducted our literature review using the Web of Science and Scopus, both of which are highly recognized databases of social science journal articles. Taking the readability of the audiences among the educational research journals into consideration, we set the subject areas as “Psychology Educational” and “Education and Educational Research” to construct a preliminary framework of educational research regarding MRI neuroimaging. To identify articles on MRI-based educational research, we used keywords based on the domain (Wang and Chen, 2009) and method (Shenton et al., 2012) of the MRI-oriented research, and the following keywords were used to search the topic: “brain or neuroimaging or neuroscience” and “MRI or DTI or ‘white matter’ or ‘gray matter’ or resting-state.” The time span was set from 2000 to 2019 to cover two decades after 2000. The document type was limited to English journal articles to ensure that the studies were of potentially more consistent quality because of the peer-review process. Finally, the subject areas of Web of Science was set as “Psychology Educational” and “Education and Educational Research” within the Social Sciences Citation Index. Forty-two articles were retrieved from the Web of Science database. In addition, 6,778 articles in the “Psychology” and “Social Science” sub-areas were retrieved from the Scopus database. Among the 6,778 articles, 25 articles were in accordance with the subject areas of “Educational Psychology” and “Education and Educational Research” of the Web of Science. Forty-seven articles were finally included after excluding 20 duplicates.

The researchers manually and systematically screened the title and abstract of the article, excluding (1) the same abbreviations but different semantics as MRI (*n* = 7); (2) no neuroimaging data (*n* = 10); and (3) cognitive neuroscience research for non-brain imaging (*n* = 5). Finally, 25 articles were included in this study. The PRISMA flow diagram is presented in Appendix 1. Besides information regarding the authors, publication year, subject, journal, and other information of these articles is provided in Appendix 2.

### Coding Procedure

The content analysis consisted of two stages. Two experts in the education and cognitive neuroscience research domain coded the 25 papers separately and discussed the content to establish coding consistency. In the first stage, the content of a selected paper was preliminarily coded based on the title and abstract of the article to specify nine subtopics: language, creativity, music, physical activity, biology learning, mathematical learning, physics learning, parenting, and personality development. According to the topic and research methods, we integrated some of these subtopics into broader topics. First, both reviewed studies in the subtopics of parenting and personality development focused on neural plasticity in longitudinal studies; therefore, the two subtopics were merged into the topic termed *brain development*. Next, the subtopics of biology learning, mathematical learning, and physics learning were classified as *science education*. Finally, the subtopics of language, creativity, music, and physical activity all involved cognitive processes; therefore, they were sorted into the third topic, *cognitive function*. In short, three major themes were formed according to the abovementioned method: cognitive function, science education, and brain development.

In the second stage, we classified the cognitive neuroscience methods according to which one the study used, namely fMRI, sMRI, and DTI. Finally, based on Erikson's eight stages of psychosocial development (Erikson, 1982) and the number in each stage among these reviewed studies, we divided the participants into four groups based on their age: latency middle childhood (7–12 years), adolescence (12–19 years), early adulthood (20–39 years), and middle adulthood (40–59 years).

## Results

### Overall Findings

Retrieved from the crucial databases such as WOS and SCOPUS from 2000 to 2019, the current study systematically reviewed 25 educational journal papers that report the use of MRI techniques. In this section, we first provide an overview of the retrieved papers in terms of publication year, domain/sub-domain, method, and participants' age level. Next, the reviewed papers are categorized into three major domains (i.e., Cognitive Function, Science Education, and Brain Development) as well as their main findings, presented in order to reveal the current state of research practice.

As shown in Table 1, two to three papers were published each year from the period of 2011–2015, while only one article appeared in the years of 2016, respectively. It is worth noting that, in 2018, seven educational studies related to neuroscience using MRI techniques were published. It would seem that this line of research has gradually drawn more researchers' attention recently.

**Table 1:** Number of reviewed studies in terms of publication year.

**Year**	***n***	**Year**	***n***
2011	3	2016	1
2012	2	2017	2
2013	2	2018	7
2014	3	2019	2
2015	3		

**Figure 1:**
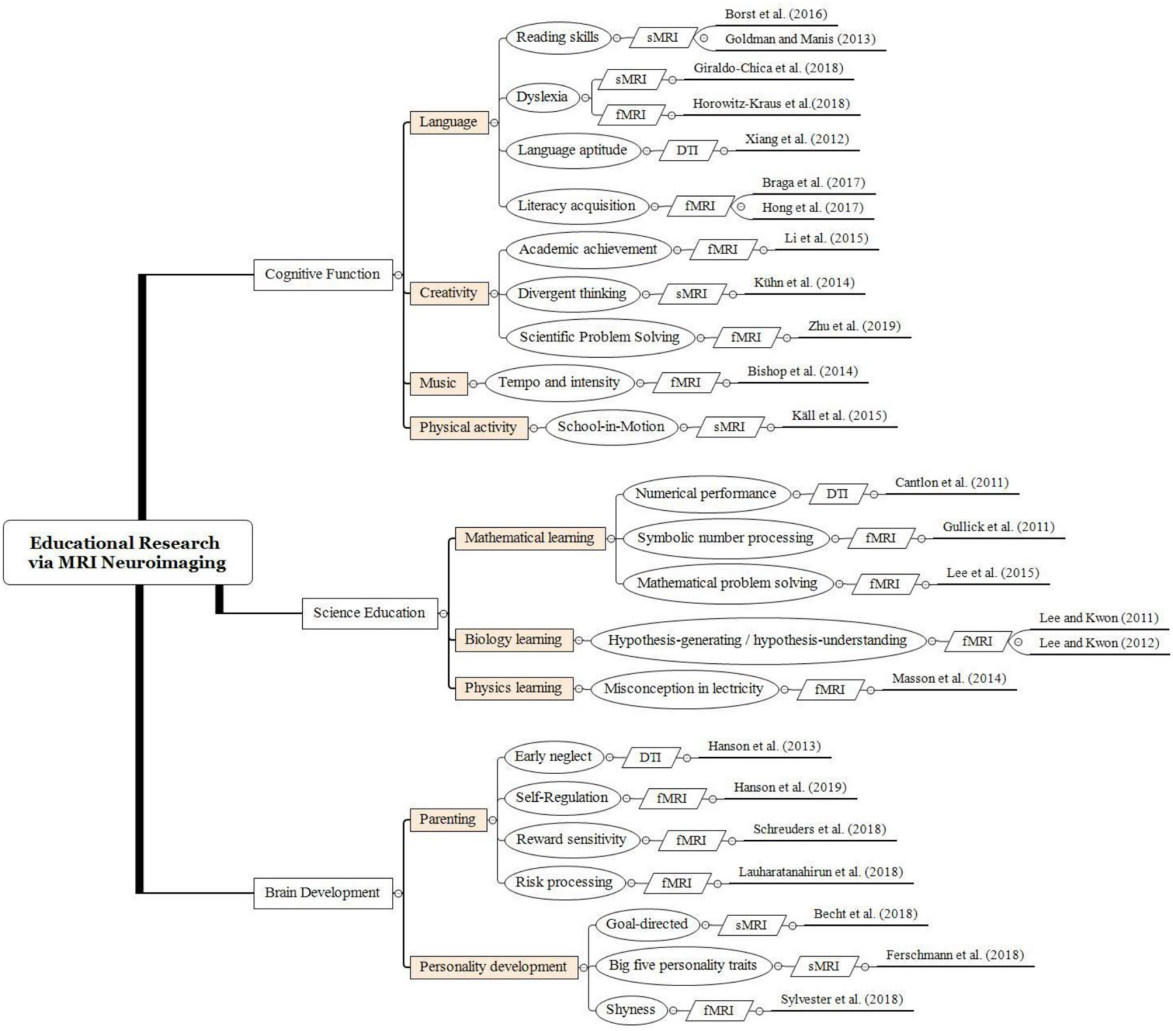
Educational research via MRI neuroimaging in the reviewed studies.

Furthermore, the reviewed studies were categorized into three domains, namely Cognitive Function, Science Education, and Brain Development. The level and category of the theoretical foundations adopted by the reviewed studies are illustrated as Figure 1 and Table 2. As presented in Figure 1 and Table 2, in general, the Cognitive Function domain (*N* = 12) was most frequently investigated by the researchers, followed by the Science Education (*N* = 6) and the Brain Development domain (*N* = 7). To be more specific, it seems that sub-domains such as Language (*N* = 7), Parenting (*N* = 4), Mathematical learning (*N* = 3), Creativity (*N* = 3), and Personality development (*N* = 3) were mostly highlighted from 2011 to 2019.

**Table 2:** Number of reviewed studies in terms of domain and sub-domain.

**Domain**	***n***	**Domain**	***n***
Cognitive Function	12	Science Education	6
Language	7	Mathematical learning	3
Creativity	3	Biology learning	2
Music	1	Physics learning	1
Physical activity	1	Brain Development	7
		Parenting	4
		Personality development	3

The reviewed studies tended to favor certain methods in each domain. As shown in Table 3, fMRI had been commonly adopted in the evaluation of cognitive function (*N* = 6) and science education (*N* = 5) domains, while sMRI had been used in the evaluation of cognitive function domain (*N* = 5). DTI has been the least applied tool to collect relevant data in the three categorized domains (*N* = 1).

**Table 3:** Number of reviewed studies in terms of domain and method.

**Domain/Method**	***N***	**Domain/Method**	***n***
Cognitive Function	12	Science Education	6
fMRI	6	fMRI	5
sMRI	5	DTI	1
DTI	1	Brain Development	7
		fMRI	4
		sMRI	2
		DTI	1

Furthermore, the distributions of age level in the three categorized domains are presented in Table 4. First, in the reviewed studies, the early adulthood group was usually involved in both the cognitive function (*N* = 6) and science education (*N* = 4) domains. In contrast, among the reviewed studies in the domain of brain development, all studies involved participants who were below 19 years of age, including latency middle childhood (*N* = 3) and adolescence (*N* = 4). It should be noted that only one study (Science Education domain) invited both children and adults to compare brain activation areas with their behavioral data.

**Table 4:** Number of reviewed studies in terms of domain and age level.

**Domain**	***n***	**Domain**	***n***
Cognitive Function	12	Science Education	6
Early adulthood	6	Early adulthood	4
Latency, middle childhood	4	Adolescence	1
Middle adulthood	2	Latency, middle childhood/early adulthood	1
		Brain Development	7
		Adolescence	4
		Latency, middle childhood	3

### The Three Categories of Educational Studies via MRI Neuroimaging

#### Cognitive Function

Twelve studies explored how cognitive function is associated with brain activities. Seven studies examined the relationships between language skills and brain mechanisms. Five papers investigated other cognitive functions: one investigated music, another physical activity, and the others creativity.

The ability to use spoken and written language has been the most remarkable of abilities for human beings (Chang and Lee, 2020). However, humans are biologically endowed to speak but not necessarily to read and write. Recent MRI neuroimaging studies have largely advanced our knowledge of how the brain evolves during the process of literacy. For example, it has been well-recognized that reading relies on a left lateralized brain network, including the inferior frontal/precentral gyri, the dorsal temporoparietal circuit, and the ventral occipitotemporal. Specifically, the visual word form area (VWFA) in the left lateral occipitotemporal sulcus (OTS) is specialized for the orthographic analysis of written words (Dehaene and Cohen, 2011). Braga et al. (2017) used fMRI to trace the changes of brain activation in VWFA for reading acquisition in an illiterate adult. Initially, the VWFA showed identical activation for processing words, faces, and checkerboards. However, the word-induced activation in VWFA increased steadily with time and positively correlated with reading performance. Reading-related responses also emerged in language-related areas of the inferior frontal gyrus (IFG) and temporal lobe. These results indicate that adult plasticity can be sufficient to induce rapid changes in brain responses to written words.

Other studies examined the relationship between brain morphology and reading ability. For example, the sulcal morphology is a qualitative feature of the brain which is determined *in utero* and is not affected by the learning process. Borst et al. (2016) reported that the left but not the right OTS's sulcal pattern predicted oral reading accuracy as measured by the number of French words correctly read in 3 min. Thus, the result may be important for the early detection of dyslexia. Other studies examined the relationship between brain morphology and reading ability. Goldman and Manis (2013) reported that print exposure accounted for unique variance in the cortical thickness of the occipitotemporal area, angular gyrus, supramarginal gyrus, opercularis, and triangularis part of the inferior frontal gyrus, beyond the variation predicted by reading skill. Individuals with more print exposure had thicker cortices within the left hemisphere reading network.

Another study (Horowitz-Kraus et al., 2018) examined the correlation between the fixation time and functional connectivity of neural circuits associated with executive functions among children with reading difficulty. The study compared the fixation time during word reading between children with reading difficulty and typical readers, and analyzed the association between the fixation time and functional connectivity of the brain involved in reading and executive functions during rest. The results showed that children with reading difficulty had longer fixation times during word reading. Also, negative functional connectivity between the primary cognitive control area (anterior cingulate cortex) and other areas associated with cognitive control (inferior frontal gyrus) in children with reading difficulty and negative functional connectivity involving the language regions (superior temporal gyrus and entorhinal cortex) in typical readers were found. The study connects eye movement patterns with executive functions and provides neurobiological support for this connection in children.

Hong et al. (2017) examined the effects of online English educational games on second language learning in Korean children by evaluating the changes in the functional connectivity of the brain. After the game, an increase in positive connections was observed between the Broca area and left frontal cortex and between the Wernicke area and left parahippocampal gyrus and right middle frontal gyrus. On the other hand, the increased connectivity between Wernicke's area and the left parahippocampal gyrus was positively correlated with the change in non-verbal pragmatic score. The results show that online English education games can enhance L2 learning and train verbal and non-verbal information systems using visual, auditory, tactile, and kinesthetic sensory methods, especially in non-verbal pragmatic skills.

Xiang et al. (2012) examined the relationship between language learning aptitude and the structural connectivity of four possible language pathways among the typical reading network (left parietal lobe, posterior temporal lobe, and the Broca's complex). Language learning aptitude is assumed to be a predominantly innate and somewhat fixed “gift” for learning languages. Results showed significant correlations between the scores of the four components of the aptitude tests and the structural connectivity of certain language pathways, that is, grammatical inferencing and the BA45 and BA46 Temporal pathways, sound-symbol correspondence and the interhemispheric BA45 pathway, and vocabulary learning and the BA47 Parietal pathway. The findings also shed some light on the specific functions served by different language pathways.

Relatively few studies have examined the relationship between the subcortical structure and reading ability. Giraldo-Chica and Schneider (2018) investigated the asymmetries in morphology, orientation, and location of the lateral geniculate nucleus (LGN) in individuals with or without dyslexia, as the LGN is the primary visual relay nucleus from the retina to the cortex and is a pivotal control structure in visual processing and attention. This study found that the LGN in dyslexia is oriented to be more parallel to the axial plane in the left in comparison to the right hemisphere. Moreover, the location of LGN showed hemispheric differences in controls, but not in dyslexia. Asymmetries in the position and morphology of the LGN are critical as the potential significance of the LGN in the magnocellular theory of dyslexia. However, it remains unclear if the anatomical difference in LGN might be caused of dyslexia.

Other cognitive function studies in educational neuroscience have focused on creativity (Kühn et al., 2014; Li et al., 2015; Zhu et al., 2019), physical activity (Käll et al., 2015), and music (Bishop et al., 2013). Regarding studies about creativity, the brain structure difference of adults with different levels of creativity was examined via the resting-state fMRI. The results underpinned that greater gray matter volume (GMV) is associated with higher creative performance, and creativity requires both hemispheres rather than the right hemisphere only. Li et al. (2015) scanned the brain structure of 22 healthy male university professors with different levels of scientific creativity. According to their academic achievement (i.e., the number and citations of publications, the number, and significance of research projects), the professors were divided into a high achievement professors group and a low achievement professors group. The results demonstrated that the regional GMV of the high achievement professors was larger than that of the low achievement professors in the left IFG, the left supplementary motor cortex, and the left anterior cingulate cortex. All the above regions are associated with goal-directed behavior, that is, behavioral planning, execution, and regulation.

Kühn et al. (2014) implemented the most used creativity tests and an MRI scan on a sample of 21 healthy adults to investigate the association between creativity and default mode network. The default mode network, a set of interconnected regions in the brain, is linked with mind-wandering and unconscious information processing. This research indicated that the correlation between the GMV of the ventromedial prefrontal cortex, a core region of the default mode network, and creativity performance is significantly positive. The current research also found a positive correlation between creative performance and the GMV of some brain regions in both hemispheres. Moreover, Zhu et al. (2019) examined the neural substrates of the representation-connection based on integrated analysis approaches, including structural [regional gray matter density (rGMD)], functional [fractional amplitude of low-frequency fluctuations (fALFF)], and resting-state functional connectivity (RSFC). The results showed that the representation-connection score was positively correlated with the rGMD and fALFF values in the bilateral lingual gyrus. In addition, the representation-connection score was negatively correlated with the rGMD in the left anterolateral prefrontal cortex. Further, functional connectivity analysis showed that representation-connection was inversely correlated with RSFC intensity between the right lingual gyrus and the left lower parietal lobe.

The effect of a curricular physical activity intervention on the structural development of the brain of elementary school children in fourth to sixth grade was examined by Käll et al. (2015). However, no significant differences in hippocampal volume, measured automatically and manually through MRI, were observed between the intervention group and control group. On the other hand, the impact of pre-task music listening on the brain function during a sport reaction performance was corroborated (Bishop et al., 2013). The tempo and intensity of music were manipulated in the study, and the results confirmed that the pre-task music listening activated the auditory cortex. During task performance, faster tempo and louder intensity produced stronger activation in brain regions associated with object recognition (inferior temporal gyrus), visual attention allocation (cuneus, inferior parietal lobule, supramarginal gyrus), and motor control (putamen). This activation may elicit heightened visual perception and motor activities. In sum, faster tempo and higher intensity of pre-task music trigger significant activations in several brain regions and consequently facilitate task performance.

#### Science Education

Six empirical studies examined the brain mechanism during science learning. Four studies explored mathematical learning, such as numerical performance and symbolic number processing. Besides, one study focused on biological learning, and the remaining three studies tested the differences in brain activation during physics learning.

First, in the mathematical learning subtopic, Gullick et al. (2011) employed fMRI to determine the possible associations to understand the influence of various cognitive factors such as working memory, non-verbal intelligence, and mathematics achievement during mathematics processing. The 17 participating adults were asked to complete several measures to understand the abovementioned cognitive factors and then performed the mathematics-based tasks (i.e., symbolic and non-symbolic comparison) with the fMRI procedure. The most significant finding derived from this study indicated that several brain regions, including the bilateral parietal, temporal, and right frontal areas, showed a positive relationship with working memory during symbolic number processing, while the bilateral parietal and right frontal areas were found to be negatively associated with working memory during non-symbolic number processing. In other words, it was argued that individual differences in working memory had stronger effects than other investigated factors during basic number processing.

In another study related to individuals' numerical performance (Cantlon et al., 2011), 18 children (averaging 6.89 years), and 14 adults (averaging 23.2 years) completed numerical judgment tasks (symbolic and non-symbolic conditions) inside the MRI scanner. DTI tractography analyses were adopted to specifically test the white matter integrity of the corpus callosum in three tracts (i.e., genu, isthmus, and splenium) with relation to numerical performance on the tasks. In general, the results indicated significant structural differences between children and adults in the interhemispheric white matter tracts connecting parietal and occipitotemporal cortices (i.e., isthmus and splenium). Moreover, the only negative relationship was found between the children's numerical judgment performance and white matter coherence, indicating that participants with poor performance tended to exhibit reduced white matter coherence in the fibers passing through the isthmus of the corpus callosum. The gained results provided distinctive evidence that the development of numerical skills among young children may link to the corpus callosum's maturity connecting the superior parietal hemispheres.

Lee et al. (2015) conducted an event-related fMRI study on mathematical problem solving. They investigated the effects of two different instructional types—studying examples and studying verbal directions. They reported that these two instructional types might induce different patterns of brain activity. Increased neural activation related to mathematical problem solving was found in the prefrontal and parietal regions while studying examples. Besides, greater activation was found in the motor and visual regions while studying the verbal directions. Nonetheless, no significant effect on neural activation was found between the two instructional types when the participants applied what they had learned to solve mathematical problems. Based on these findings, we conclude that instructional types may have little effect on the execution process of solving mathematical problems.

Second, in the biology learning subtopic, Lee and Kwon (2011) gathered behavioral and fMRI data from 60 male adults, administered two distinct yet related biological tasks, hypothesis-understanding and hypothesis-generating, and compared the neural network activation areas of the two tasks. Based on the functional connectivity network models of the two tasks, the results showed that the participants displayed different neural networks during each task. That is, for the hypothesis-generating process, all of the activated regions were left lateralized including the frontal, temporal, limbic, occipital, and sublobar lobes. On the contrary, the activation regions for the hypothesis-understanding processing were mainly the right frontal, parietal, and sublobar lobes. It is noteworthy that both the left and right corpus callosum (i.e., sublobar lobe) were activated during hypothesis-understanding, suggesting that this process requires interconnections between the two hemispheres to understand the semantic information. In sum, the findings suggested that the two hypothesis processes do not occur in the same neural network and should be trained separately.

Lee and Kwon (2012) also examined whether constant scientific reasoning training could alter brain activation regions and networks. They designed two parallel 12-week training program; while one required the participants to actively generate a hypothesis (hypothesis-generating), the other asked the participants to passively understand a hypothesis (hypothesis-understanding). A total of 14 high school participants were randomly assigned to either group. The participants were scanned with fMRI both before and after the training program in order to examine whether changes in brain activity occurred during the training programs. The results indicate that the hypothesis-generating group showed significant changes in their hypothesis generating ability as well as in their neural network associated with hypothesis-generating. In contrast, the hypothesis-understanding group did not achieve a significant improvement in their hypothesis-generating ability, but only strengthened the neural network associated with hypothesis-understanding. The findings of this study successfully confirmed that constant training could alter brain activity and the neural network.

Lastly, in the physics learning subtopic, Masson et al. (2014) conducted an fMRI study to examine whether misconceptions coexist with or are replaced by scientific conceptions in human brains. In their experiment, they asked both expert and novice students to evaluate the correctness of electric circuits representing scientific concepts and misconceptions. The results indicated that, while evaluating the electric circuits representing misconceptions, the expert students showed greater brain activity in regions associated with inhibition, such as the ventrolateral prefrontal cortex, the dorsolateral prefrontal cortex and the anterior cingulate cortex. Accordingly, the authors concluded that misconceptions might not be erased or replaced by scientific conceptions since the single argued difference between congruent and incongruent was the presence of a distractor, but it still coexisted with scientific conceptions. Experts thus need to inhibit their misconceptions in order to engage in scientific thinking and reasoning.

#### Brain Development

Seven longitudinal studies examined brain changes in the brain development section. Four studies focused on the effectiveness of parenting, and three explored personality-related topics.

In the parenting subtopic, Hanson et al. (2013) collected DTI data in children exposed to early neglect and children with typical development. They particularly focused on DTI derived measures of white matter organization: fractional anisotropy. Fractional anisotropy describes the directionality of water diffusion and is regulated by the white matter's microstructural properties, including fiber density, axonal diameter, and myelination. The authors found that early neglected children had lower white matter directed tissue in the prefrontal cortex and the directional organization in the white matter bundle connecting the temporal lobe and prefrontal cortex. Individual differences in the white matter microstructure of the fractional anisotropy index were associated with poorer neurocognitive performance among the neglected children. Hanson et al. (2019) examined how a prevention program can influence behavior during childhood and brain function in adulthood using longitudinal assessments. This program aimed to improve participants' self-regulation at the age of 11 years. As adults, these same individuals underwent functional MRI (age = 24.88 years; intervention group *n* = 44; control group *n* = 49). Compared with the control group, the intervention group showed increased functional connectivity between the hippocampus and ventromedial prefrontal cortex (a brain area involved in social-emotional functions and self-regulation). These results show an association between the neurobiological and psychosocial signs of risk and resilience.

Another study tested how the ventral striatum response to rewards develop across adolescence and early adulthood and how individual differences in state and trait level reward sensitivity are related to these changes using fMRI with an accelerated longitudinal design with three time points. Each time point was separated by 2 years (Schreuders et al., 2018). They examined the developmental trajectory of nucleus accumbens activation associated with rewards in the 8–29 age group, and how the behavioral traits and trait level reward sensitivities associated with these changes. They found that nucleus accumbens activities can reward peak periods in mid adolescence, and that differences in the development of self-reported motivational rewards and winning direct pleasure contributed to these changes.

In a sample of 167 healthy adolescents, Lauharatanahirun et al. (2018) examined how family environmental factors—parental monitoring and family chaos—might be related to the development of insular risk related processing (i.e., change scores of insula activation between Time 1 and Time 2), which is a candidate neural mechanism for adolescent risk behavior. Higher parental knowledge levels were associated with higher levels of island cortical activation in a low-chaotic environment, but not in a high-chaotic environment. A statistical moderating effect of family chaos on the link between parental knowledge and adolescent risk taking was found. This suggested that parental knowledge as perceived by adolescents played an important role in insular risk related processing in the adolescent brain, and that the beneficial effects of parental knowledge can be diminished by the chaos within home environments.

In addition, in the personality development subtopic, teaching, and Learning Activities not only change the external performance of students, but also affect their personality traits. Personality development in each stage of the life span is closely related to education (Erikson, 1982). In three separate studies, Becht et al. (2018) investigated self-reported and neural processes underlying adolescents' identity. They combined a large scale questionnaire and a structural brain development study. They applied a two-step approach to examine the longitudinal association between brain regions, goal orientation, and identity. In the first step, they showed that the individual differences in the development trajectory of the self-reported goal predicted later identity. These findings were partially replicated in the second longitudinal teen sample. In the second step, they extended these self-reported findings to neural levels by showing how individual differences in initial levels and changes in nucleus accumbens and right prefrontal cortex GMV predicted later adolescent status. In this study, the identities of the adolescents were predicted according to self-reported goal pursuits and developmental trajectories of structural brain regions. Adolescents with higher goals and volume of nucleus accumbens reported a stronger identity and less uncertainty about these commitments. Also, adolescents with a higher prefrontal cortex and more protracted prefrontal cortex volumes reported greater reflection on their identity commitments.

The other study investigated the relations between personality traits and cortical thickness and surface area in children and adolescents (Ferschmann et al., 2018). The results showed that all five major personality traits were associated with longitudinal structural cortical development, both in cortical thickness and surface area, occurring across adolescence. This study enriched existing knowledge by providing insights into the ontogeny and temporal dynamics of these relations by examining them in younger and longitudinal samples. Furthermore, exploring these associations in younger samples may provide information about the broader development issues because personality traits not only predict major life outcomes but also individual levels of general functioning. The study results suggested that individual differences in personality traits might be partially related to adolescent cortical maturation and imply a developmental origin for personality-brain relations previously observed in adults.

Finally, a longitudinal study examined the relationship between the changes in functional brain network connectivity and variation in temperamental shyness over early adolescence (measured between 7.8 and 15.2 years) (Sylvester et al., 2018). The results showed that the changes in functional connectivity in the default mode network during rest in early adolescence were associated with temperamental shyness. After controlling for the lifetime history of social phobia, shyness remained significantly associated with the trajectory of functional connectivity in the default mode network. Compared with subjects with high temperamental shyness, subjects with low temperamental shyness showed a more negative slope, which indicates that subjects with low temperamental shyness had more of a decrease in connectivity in the default mode network during early adolescence. Besides, no significant association was found between age and the level of shyness in the current study.

## Discussion

As our study results show, 25 MRI neuroimaging studies on education-related topics since 2000 were reviewed and divided into three domains: cognitive function, science education, and brain development. In these studies, the theme of language acquisition has received the most attention (i.e., Xiang et al., 2012; Goldman and Manis, 2013; Borst et al., 2016; Braga et al., 2017; Hong et al., 2017; Giraldo-Chica and Schneider, 2018; Horowitz-Kraus et al., 2018), followed by mathematical learning (i.e., Cantlon et al., 2011; Gullick et al., 2011; Lee and Kwon, 2012; Lee et al., 2015). In research method, fMRI is generally applied to all three domains, while sMRI is only applied to the two domains of cognitive function and brain development. Although DTI has applications in all three domains, the number of empirical studies is relatively small. Thus, in educational research, fMRI was the main method, and DTI was relatively rare. Furthermore, regarding the age level of participants, in the cognitive function domain, the age distribution of participants was relatively wide (7–12, 20–39, and 40–59 years). In the brain development domain, the participants were relatively young (7–12, 12–19 years), and in the science education domain, the participants were mostly in early adulthood (20–39 years). This showed that studies on different topics may have participants in specific age groups. Meanwhile, the number of studies aiming to examine brain changes after learning has grown significantly in recent years (i.e., Hanson et al., 2013; Becht et al., 2018; Ferschmann et al., 2018; Lauharatanahirun et al., 2018; Schreuders et al., 2018; Sylvester et al., 2018). In short, MRI or DTI can provide images of the brain activation during specific tasks, or of the brain structure in a resting state. The foci of discussions presented in the reviewed studies has gradually shifted from exploring the basic cognitive function or the specific discipline learning associated with the brain mechanisms, to examining the effectiveness of the intervention by the neural plasticity. These results reflected the educational neuroscience deepens our understanding of cognitive processes during the teaching and learning from neural and behavioral data (Howard-Jones et al., 2016).

First, the reviewed studies on the cognitive function topic were divided into two main parts: language and other functions (i.e., creativity, music). In the language subtopic, writing systems are a recent cultural invention and vary substantially in terms of how the visual codes represent spoken language. Studies across writing systems have identified a preexisting neural architecture in the left hemisphere for reading, including the IFG, the dorsal temporoparietal circuit, and the ventral occipitotemporal area. To be more specific, the acquisition of reading ability may be considered as a special case of perceptual-learning experience, and the “visual word form area” located in the left lateral occipitotemporal sulcus appears to be specialized for the orthographic analysis of written words (Dehaene and Cohen, 2011). Meanwhile, writing systems were invented to represent spoken words and their corresponding meanings. There is now strong evidence suggesting that there are two neural pathways for reading. The dorsal pathway underpins phonological mediated reading involving brain activities in the temporoparietal junction and IFG. The ventral pathway temporal gyrus to the IFG is responsible for the direct mapping from print to meaning. Children with difficulties in learning to read often show reduced or absent activation in the left lateralized neural network when reading. The studies of brain morphology, such as a white matter organization, also reveal weaker connections among the neural circuit of reading in those with dyslexia, and these measures correlate positively with reading achievement. Nowadays, the diagnosis of dyslexia is typically made in children who are starting formal schooling and who fail in their reading performance. However, the remediation of dyslexia is much more likely to be successful when conducted on children begging or before learning to read. The functional and structural neural signatures will be of great assistance in the early identification and remediation of atypical reading development. However, there is still a big gap between fundamental research of cognitive neuroscience and practical applications in education. Further work to link the brain data and pedagogical innovations promises to be a particularly important research field for future education and neuroscience.

Moreover, the findings of other studies on cognitive function indicate a bidirectional relationship between cognitive function and brain structure. For instance, individuals with greater GMV may be more creative, and creativity practice or training may cause GMV change (Kühn et al., 2014; Li et al., 2015). Future studies can implement longitudinal studies or educational interventions to better understand the relationship. Furthermore, since the effect of physical activity intervention on hippocampal volume was found in adolescents but was not seen in Käll et al.'s (2015) study, more efforts should be made in the future to investigate the role of age in the structural development of the brain. Regarding the impact of cognitive function on brain function, Bishop et al.'s (2013) study revealed that pre-task music listening generates significant brain activations during task performance. The author recommends that pre-task music listening could be applied in educational settings, and that official competition could improve task performance.

Second, five fMRI studies on science education were conducted to explore the relationships between some domain-specific cognitive functions (e.g., conceptual change, scientific discovery, and numerical processing) and their corresponding brain regions. These relevant studies manipulated the abovementioned cognitive functions into different levels (such as misconception vs. cognitive inhibition in conceptual change, and hypothesis-generation vs. hypothesis-understanding in scientific discovery), and successfully identified different brain regions associated with the different levels of cognitive functions. Future studies of science and mathematics education may focus on other major cognitive functions relevant to science and mathematics learning, such as scientific and mathematical inquiry, argumentation, and modeling, to explore their corresponding brain regions (Lawson, 2003; Anderson, 2009). Since these research topics have been highly valued and emphasized in recent years by researchers (e.g., Lin et al., 2019), this sort of study would be beneficial because the derived findings might inform science and mathematics educators whether these major cognitive functions have their distinctiveness and whether each cognitive function can be categorized into different levels. In addition, to what extent domain specificity has an effect on relevant cognitive functions deserves further examination. In other words, it is of value to examine whether the neural differences between different levels of cognitive functions in a certain subject area (such as the misconception/cognitive inhibition in physics) could also be found in other subject domains (such as chemistry and biology). It would be beneficial to further justify whether specific instructional approaches can be effective across different subject domains. The findings of these future endeavors can also yield a more coherent and predictive educational neuroscience theory on how science and mathematics learning occurs from a finer grained perspective (e.g., Anderson, 2014).

Last, seven longitudinal studies on brain development found that education-related factors (e.g., family environment and parenting style) not only changed individual behavior (e.g., risk processing) and traits (e.g., personality, self-identity, and shyness) but also affected the brain structure (Becht et al., 2018; Ferschmann et al., 2018; Lauharatanahirun et al., 2018; Schreuders et al., 2018). These results revealed that the neural plasticity could be used as an emerging indicator to evaluate the effectiveness of the intervention. To make the structural brain changes a more objective and accurate reference, there are still some directions for the future research. First, it will be essential to include multiple measures that represent the real-life behavior of the learners. More accurate connections between neural activities and cognitive functions would be established if more realistic external criteria were used. Second, in the teaching process, the change and bidirectional associations between structural brain and cognitive function should be tested concurrently to investigate possible developmental order. Third, future studies may provide more time-point surveys to examine the stability of the brain changes from the training.

Based on the topic of our reviewed papers, we found an increase in the number of studies testing the effectiveness of training or development via the neural plasticity approach; this finding indicates that researchers have begun to focus on the effectiveness of education from the perspective of brain mechanisms. Executing educational neuroscience research relies on the cooperation between experts in education, psychology, and cognitive neuroscience. This shows that the field of educational neuroscience is in its emergent phase; moreover, close and interdisciplinary collaboration is indispensable in educational neuroscience research.

Our review has some limitations. Only 25 articles from the “Psychology Educational” and “Education and Educational Research” subject areas of the Web of Science and Scopus databases met our inclusion criteria. Although the Web of Science and Scopus are both highly recognized databases containing most journal articles, it may be difficult to avoid publication bias. Besides, the number of selected articles is less than in other fields, for example, psychiatry (Mouchlianitis et al., 2016) and medicine (Andryszak et al., 2017). In addition, the domains of our review–cognitive function, science education, and brain development–are insufficient to represent all educational issues.

Finally, we call for attention to the issue of educational practice in future studies. In this review, we propose a framework to show how MRI neuroimaging methods have been applied to educational studies and what studies might gain more understanding of student learning. However, the current studies only end with understanding the association between cognitive function and the brain mechanisms, or exploring the brain changes through training. Few studies have discussed how to apply these results from a neuroimaging approach to enhance teaching activities or pedagogy in specific disciplines. Although a brain-based curriculum and teaching have been proposed (Wang and Chen, 2009), the results of empirical studies are still lacking. It is worth noting that the results of MRI neuroimaging research mostly came from indirect inferences (Bowers, 2016). Cautious and conservative application of relevant results to educational practice is needed. Additionally, as suggested previously, since collaborative studies across disciplines and expertise are needed in the future, the positive communication between researchers and teachers can immediately upgrade the information of the brain development associated with learning. For instance, the development of the prefrontal cortex is believed to play a crucial role in the storm and stress of adolescence (Casey et al., 2010; Tottenham and Galván, 2016). This finding suggests that teachers need to take more heed of the influences of emotion on learning. The close bidirectional cooperation between researchers and teachers will create a win-win situation and maximize the practical value of the MRI neuroimaging studies in educational research. It is hoped that such empirical research associated with educational theory and practice will appear in the near future.

## Data Availability Statement

The original contributions presented in the study are included in the article/Supplementary Material, further inquiries can be directed to the corresponding author/s.

## Author Contributions

C-LW collected and analyzed the data. C-LW, T-JL, G-LC, C-YL, HL, and M-JT assisted in literature review and wrote the initial draft of the manuscript. PP and C-CT monitored and supervised all aspects of the study. All authors approved the final version of the paper.

## Conflict of Interest

The authors declare that the research was conducted in the absence of any commercial or financial relationships that could be construed as a potential conflict of interest.
