# Supplementary material for: A Systematic Review of MRI Neuroimaging for Education Research
Source: Front Psychol. 2021 May 20;12:617599. doi: 10.3389/fpsyg.2021.617599 (PMC8174785; doi:10.3389/fpsyg.2021.617599)
Supplement: Supplementary file 1 [file Table_1.docx]

**Appendix 1**. PRISMA flow diagram

**Identification**

**Screening**

**Included**

Records removed *before screening*:

Duplicate records removed (n = 20)

Records marked as ineligible by automation tools (n = 0)

Records removed for other reasons (n = 0)

Records excluded (n = 0)

Reports sought for retrieval (n = 47)

Reports not retrieved (n = 0)

Reports assessed for eligibility

(n = 25)

Records identified through database searching with the keywords “brain or neuroimaging or neuroscience” and “MRI or DTI or white matter or gray matter or resting-state.”

Web of Science (n = 42)

Scopus (n = 25)

Reports excluded:

1. The same abbreviations but different semantics as MRI (n = 7)
2. Not neuroimaging research (n =10)
3. Not MRI research (n = 5)

Studies included in review (n = 25)

Reports of included studies (n = 25)

Records screened (n = 47)
