# Supplementary material for: A Systematic Review of MRI Neuroimaging for Education Research
Source: Front Psychol. 2021 May 20;12:617599. doi: 10.3389/fpsyg.2021.617599 (PMC8174785; doi:10.3389/fpsyg.2021.617599)
Supplement: Supplementary file 2 [file Table_2.docx]

**Appendix 2**. Analysis of basic information in each reviewed study

| **ID** | **Author** | **Year** | **Title** | **Journal** | **Domain** | **sub-Domain** | **Research Method** | **Age-level** |
| --- | --- | --- | --- | --- | --- | --- | --- | --- |
| 1 | Borst et al. | 2016 | Early Cerebral Constraints on Reading Skills in School-Age Children A MRI Study | Mind, Brain, and Education | Cognitive Function | Language | sMRI | latency, middle childhood |
| 2 | Goldman and Manis | 2013 | Relationships Among Cortical Thickness, Reading Skill, and Print Exposure in Adults | Scientific Studies of Reading | Cognitive Function | Language | sMRI | early adulthood |
| 3 | Giraldo‐Chica et al. | 2018 | Hemispheric asymmetries in the orientation and location of the lateral geniculate nucleus in dyslexia | Dyslexia | Cognitive Function | Language | sMRI | early adulthood |
| 4 | Horowitz-Kraus et al. | 2018 | Longer Fixation Times During Reading Are Correlated With Decreased Connectivity in Cognitive-Control Brain Regions During Rest in Children | Mind, Brain, and Education | Cognitive Function | Language | fMRI | latency, middle childhood |
| 5 | Xiang et al. | 2012 | The Structural Connectivity Underpinning Language Aptitude, Working Memory, and IQ in the Perisylvian Language Network | Language Learning | Cognitive Function | Language | DTI | early adulthood |
| 6 | Braga et al. | 2017 | Tracking Adult Literacy Acquisition With Functional MRI: A Single-Case Study | Mind, Brain, and Education | Cognitive Function | Language | fMRI | middle adulthood |
| 7 | Hong et al. | 2017 | English language education on-line game and brain connectivity | ReCALL | Cognitive Function | Language | fMRI | latency, middle childhood |
| 8 | Li et al. | 2015 | Brain Structure and Resting-State Functional Connectivity in University Professors with High Academic Achievement | Creativity Research Journal | Cognitive Function | Creativity | fMRI | middle adulthood |
| 9 | Kühn et al. | 2014 | The Importance of the Default Mode Network in Creativity: A Structural MRI Study | Journal of Creative Behavior | Cognitive Function | Creativity | sMRI | early adulthood |
| 10 | Zhu et al. | 2019 | Individual Differences in Brain Structure and Resting Brain Function Underlie Representation-Connection in Scientific Problem Solving | Creativity Research Journal | Cognitive Function | Creativity | fMRI | early adulthood |
| 11 | Bishop et al. | 2014 | Tempo and intensity of pre-task music modulate neural activity during reactive task performance | Psychology of Music | Cognitive Function | Music | fMRI | early adulthood |
| 12 | Käll et al. | 2015 | Effects of a Curricular Physical Activity Intervention on Children's School Performance, Wellness, and Brain Development | Journal of School Health | Cognitive Function | Physical Activity | sMRI | latency, middle childhood |
| 13 | Cantlon et al. | 2011 | Inter-parietal white matter development predicts numerical performance in young children | Learning and Individual Differences | Science Education | Mathematical learning | DTI | latency, middle childhood / early adulthood |
| 14 | Gullick et al. | 2011 | Individual differences in working memory, nonverbal IQ and mathematics achievement and brain mechanisms associated with symbolic and non-symbolic number processing | Learning and Individual Differences | Science Education | Mathematical learning | fMRI | early adulthood |
| 15 | Lee et al. | 2015 | Learning From Examples Versus Verbal Directions in Mathematical Problem Solving | Mind, Brain, and Education | Science Education | Mathematical learning | fMRI | early adulthood |
| 16 | Lee and Kwon | 2011 | Why traditional expository teaching–learning approaches may founder? An experimental examination of neural networks in biology learning | Journal of Biological Education | Science Education | Biology learning | fMRI | early adulthood |
| 17 | Lee and Kwon | 2012 | Learning-Related Changes in Adolescents' Neural Networks During Hypothesis-Generating and Hypothesis-Understanding Training | Science & Education | Science Education | Biology learning | fMRI | adolescence |
| 18 | Masson et al. | 2014 | Differences in Brain Activation Between Novices and Experts in Science During a Task Involving a Common Misconception in electricity | Mind, Brain, and Education | Science Education | Physics learning | fMRI | early adulthood |
| 19 | Hanson et al. | 2013 | Early Neglect Is Associated With Alterations in White Matter Integrity and Cognitive Functioning | Child Development | Brain Development | Parenting | DTI | latency, middle childhood |
| 20 | Hanson et al. | 2019 | A Family Focused Intervention Influences Hippocampal-Prefrontal Connectivity Through Gains in Self-Regulation | Child Development | Brain Development | Parenting | fMRI | latency, middle childhood |
| 21 | Schreuders et al. | 2018 | Contributions of Reward Sensitivity to Ventral Striatum Activity Across Adolescence and Early Adulthood | Child Development | Brain Development | Parenting | fMRI | adolescence |
| 22 | Lauharatanahirun et al. | 2018 | Neural Correlates of Risk Processing Among Adolescents Influences of Parental Monitoring and Household Chaos | Child Development | Brain Development | Parenting | fMRI | adolescence |
| 23 | Becht et al. | 2018 | Goal‐Directed Correlates and Goal-Directed Correlates and Neurobiological Underpinnings of Adolescent Identity: A Multimethod Multisample Longitudinal Approach | Child Development | Brain Development | Personality development | sMRI | adolescence |
| 24 | Ferschmann et al. | 2018 | Personality Traits Are Associated With Cortical Development Across Adolescence: A Longitudinal Structural MRI Study | Child Development | Brain Development | Personality development | sMRI | adolescence |
| 25 | Sylvester et al. | 2018 | Shyness and Trajectories of Functional Network Connectivity Over Early Adolescence | Child Development | Brain Development | Personality development | fMRI | latency, middle childhood |
